# Angiomatosis Occurring in an Uncommon Location and Age Group: A Case Report and Literature Review

**DOI:** 10.7759/cureus.52154

**Published:** 2024-01-12

**Authors:** Ebenezer O Fatunla, Babatope L Awosusi, Francis A Onakpoma

**Affiliations:** 1 Department of Pathology, University College Hospital, Ibadan, NGA; 2 Department of Pathology, King Khaled Hospital, Al Majma'ah, SAU; 3 Department of Pathology, Terre Haute Regional Hospital, Terre Haute, USA

**Keywords:** vascular neoplasm, upper back, vascular lesion, diffuse haemangioma, angiomatosis

## Abstract

Angiomatosis is a rare benign vascular malformation. The lesion has a highly infiltrative nature and a high recurrence rate, making it easily misdiagnosed as malignancy. Therefore, diagnosis is best made based on a combination of clinical, radiological, and histological features. This case presentation is unique because aside from the fact that the lesion is rare, it was seen in an uncommon age and location. Occurrence in such location has not been documented, making this presentation the first of its kind. A 29-year-old male presented with a swelling on the right side of the back, reportedly present for the past six years. The swelling was painless, with a history of progressive increase in size. Examination at presentation revealed a fairly round mass located on the back, 5 cm below the right scapula. He underwent a wide local surgical excision with the material sent for histopathological evaluation. Based on the morphologic features, a definitive angiomatosis diagnosis was made. Our patient had complete surgical excision with histologically confirmed free margins and no recurrence after eight months of post-operative follow-up.

## Introduction

Angiomatosis, also known as diffuse haemangioma, is a rare vascular lesion [1]. In 1992, Rao VK and Weiss SW analyzed 51 cases of soft tissue angiomatosis; since then, only a few other cases have been reported [1]. According to a systematic literature review conducted by Najm A et al. using the PubMed and Medline databases, only 48 cases of cystic angiomatosis have been reported so far [2]. Angiomatosis is a benign, yet clinically extensive vascular malformation that primarily affects children [3]. It typically involves a large segment of the body in a contiguous pattern without a dermatomal distribution [3]. The lesion is highly infiltrative and has a high recurrence rate, making it easily misdiagnosed as a malignancy [4]. Therefore, diagnosis is best made based on a combination of clinical, radiological, and histological features [4]. This presentation is unique not only because the lesion is rare but also because this index case was seen in an uncommon age group and location.

## Case presentation

**Figure 1:**
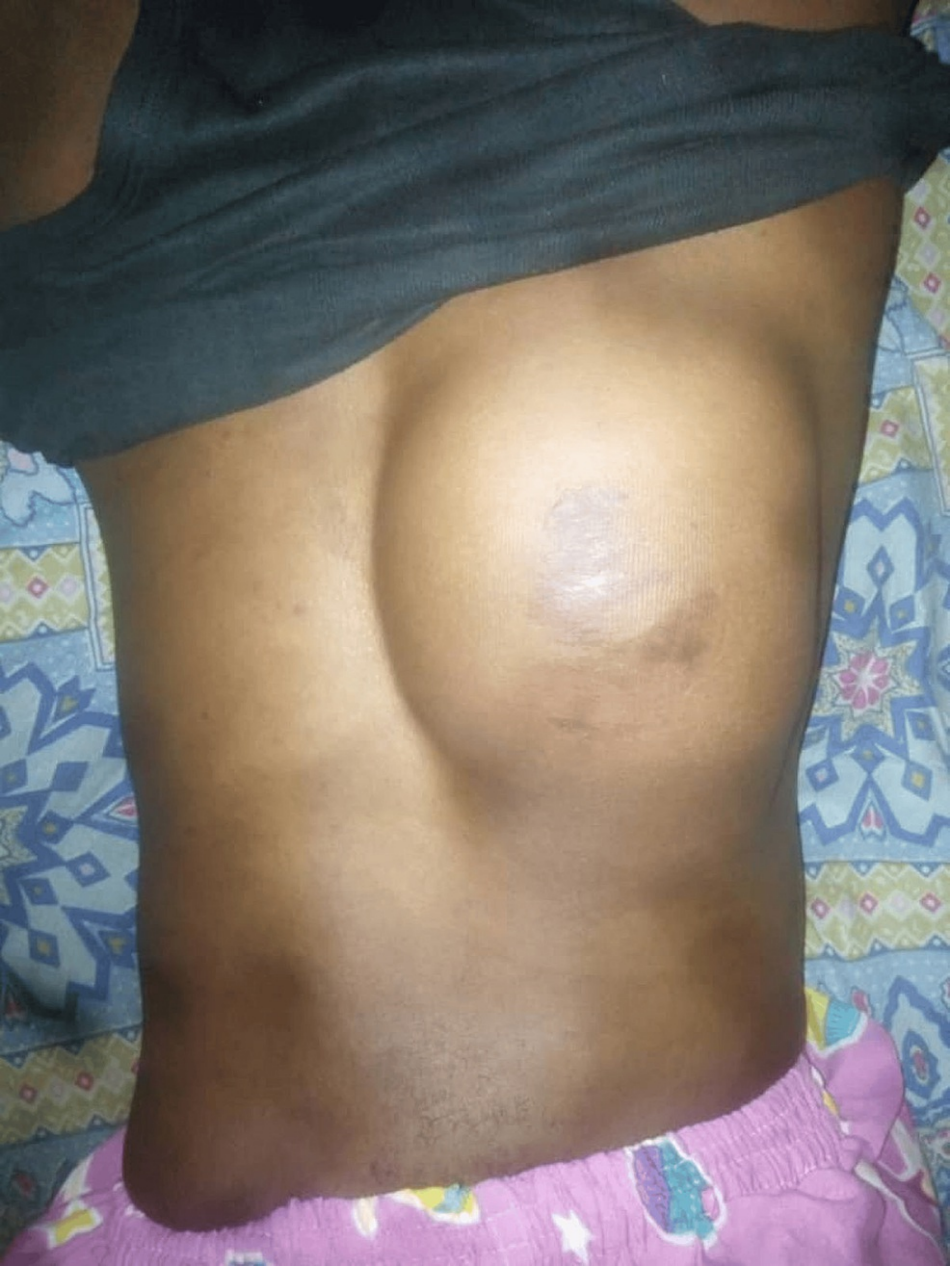
Clinical photograph displaying multiple scars overlying a fairly round, huge mass. The mass, measuring 18cm x 12cm, is located on the right upper back and abuts the midline.

A 29-year-old male presented with a swelling on the right side of the back, reportedly for six years’ duration. The swelling was painless, with a history of progressive increase in size. An initial attempt at surgical excision two years after the onset of symptoms at an outside hospital was aborted because of excessive intra-operative hemorrhage. Examination at presentation revealed a fairly round mass located in the back, 5 cm below the right scapula and abutting on the midline; it measured 18cm x 12cm x 6cm in dimension (Figure 1).

**Figure 2:**
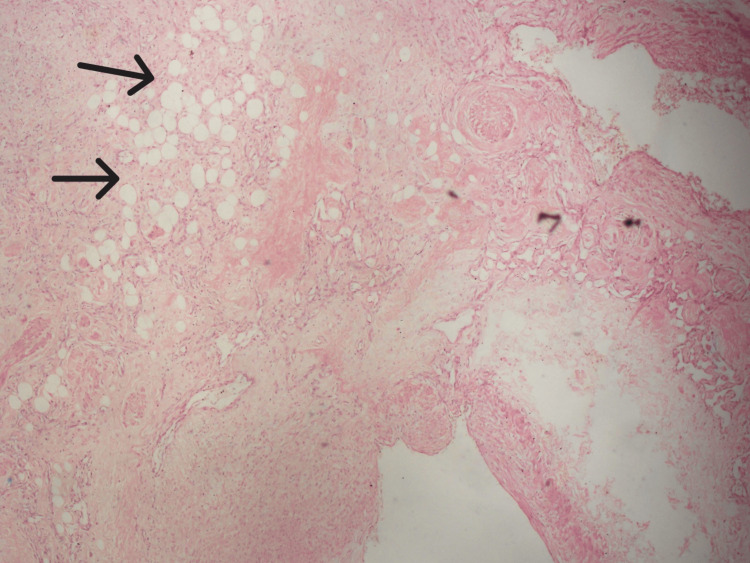
Photomicrograph depicting the proliferation of variably sized blood vessels intermixed with adipocytes (indicated by black arrows). Stained with H&E, at x200 magnification.

The overlying skin had multiple scars. The mass was attached to underlying structures; it was not tender to palpation and had no differential warmth. It was firm and non-compressible. Other physical examination findings were unremarkable. A provisional clinical diagnosis of soft tissue sarcoma was made. An ultrasound examination revealed an oval, highly vascular, poorly defined hypoechoic lesion on the right side of the back, extending from the subcutaneous tissue to the deep muscles of the back. He underwent a wide local surgical excision, and the biopsy was sent for histopathological examination. Microscopic findings showed a benign vascular lesion composed of various haphazardly arranged large venous, cavernous, and capillary-sized vessels lined by bland endothelial cells, with infiltration into the fibrocollagenous stroma, skeletal muscles, and adipocytes (Figure 2).

**Figure 3:**
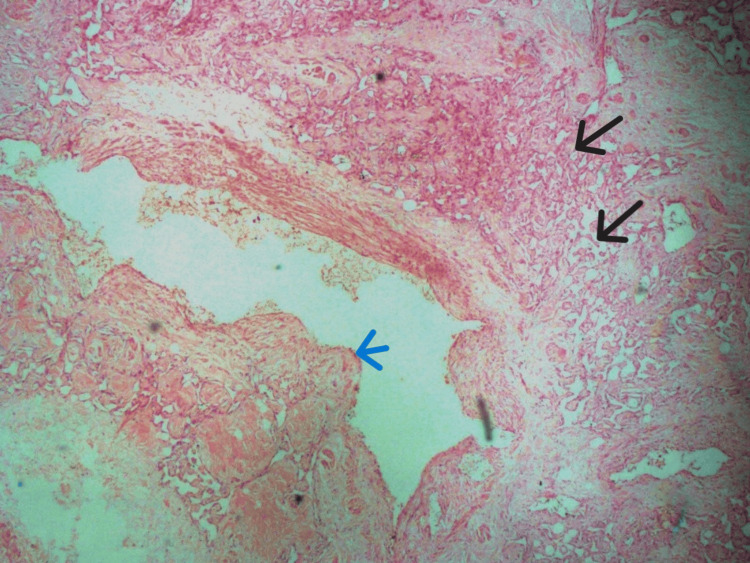
Photomicrograph displaying a large central thick-walled vein with herniation of a wall segment into the lumen (indicated by a blue arrow). Additionally, multiple small vessels are visible in the wall of the large vein (indicated by black arrows). Stained with H&E, at x400 magnification.

The venous component of the lesion is variably thick-walled with areas of wall herniation into the vascular lumen. Within the walls of these veins are lobules of clustered capillaries (Figure 3).

**Figure 4:**
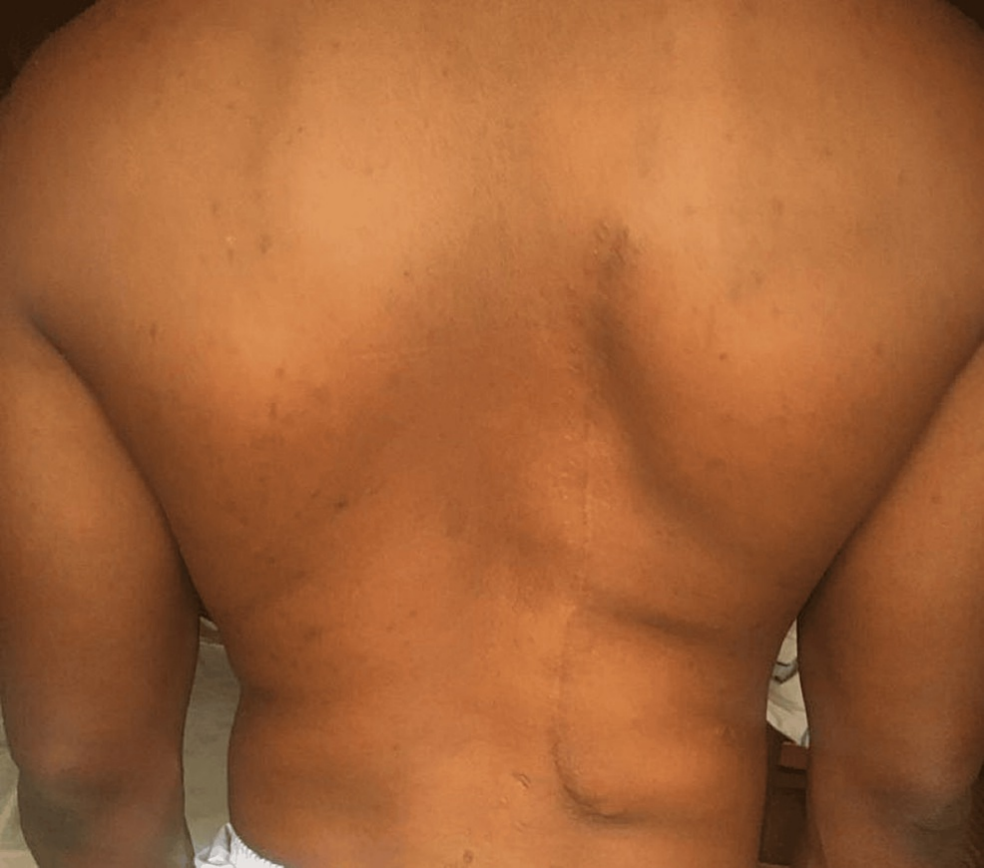
Back of the patient during post-operative follow-up at the surgical outpatient clinic.

The fibrocollagenous stroma is variably hyalinized in areas. Based on the morphologic features, a definitive angiomatosis diagnosis was made. All the margins of resection were free of the lesion. The patient had an unremarkable post-operative hospital recovery, and he is currently being followed up on an outpatient basis (Figure 4).

No adverse event or recurrence of the lesion has been reported. 

## Discussion

Angiomatosis has been known by several names in the past. 'Infiltrative angiolipoma' was given to subtypes having abundant fat, while 'intramuscular haemangioma' was used for muscle-only predominant subtypes [3]. It is composed of haphazardly arranged large venous, cavernous, and capillary-sized blood vessels lined by bland endothelial cells. Typically, it involves multiple tissue planes such as skeletal muscle, mature fat, fibrous tissue, and rarely, bone [3]. Angiomatosis is commonly seen in the first two decades of life [3]. However, our case presents a unique occurrence in a male patient in his third decade. The development of this lesion may be either congenital or acquired [4,5]. Angiomatosis has been linked with several syndromes, including Sneddon's syndrome, Klippel-Trenaunay-Weber syndrome, Gorham syndrome, and most notably Von-Hippel-Lindau syndrome [4,6,7]. Common acquired causes of angiomatosis include trauma, arteriovenous shunts for chronic hemodialysis, and infections caused by the HIV and Bartonella species [4,6,7]. In this index patient, there were no symptoms suggestive of any syndromic association, nor were any of the documented etiological factors present, which makes the case even more interesting. Angiomatosis has been reported in various locations, including but not limited to the breast, mandible, maxilla, lips, and long bones [5,6,8,9]. Some cases involving bone angiomatosis also show visceral involvement, affecting organs such as the lungs, liver, and spleen [10]. Instances of angiomatosis in the heart, larynx, neck, and mediastinum have been documented as well [11]. The first case of paranasal sinus angiomatosis was reported in 2013 [12]. The location of this index case is the upper back, which, to the best of our knowledge, based on a literature search, has not been previously documented. This makes the presentation unusual and potentially the first of its kind.

Several differential diagnoses of angiomatosis can be considered due to its morphology and infiltrative pattern, including angiomyolipoma, angiolipoma, glomangiomatosis, and intramuscular lipoma. Angiolipoma is typically well-circumscribed; angiomyolipoma exhibits a variable admixture of blood vessels, smooth muscle, and adipocytes, and infiltrating lipoma consists of lobules of mature adipocytes and muscle [3]. In this index case, these three close differentials were successfully ruled out based solely on morphology. Sometimes, due to its infiltrative nature, angiomatosis can be easily misdiagnosed as an atypical vascular lesion (AVL) or a low-grade malignant angiosarcoma [13]. In cases where differential diagnosis is particularly challenging, the Ki-67/MIB1 proliferative index and MYC testing may be employed to resolve the dilemma. However, this lesion does not possess malignant potential [4]. Interestingly, this index case, which was eventually histologically confirmed as angiomatosis, was initially thought to be a soft tissue sarcoma clinically, with ultrasound suggesting invasion. About 90% of patients will experience local recurrence following surgery [4]. The treatment of choice is radiotherapy or immunotherapy using interferon, and in localized cases, a repeat attempt at complete resection may be performed [4,5]. Complete resection was done in this index case, and all the resection margins were histologically confirmed to be free of the lesion. There were no post-operative complications. Our patient is currently on follow-up eight months postoperatively and has no clinical feature of recurrence.

## Conclusions

Angiomatosis is a benign lesion that could mimic malignancy. Clinical, radiological, and pathological features are necessary to make a definitive diagnosis. Though commonly seen in the first two decades of life, it is not exclusive to this age group, as we just reported a case occurring in the late third decade of life.
